# Unicorn, Hare, or Tortoise? Using Machine Learning to Predict Working Memory Training Performance

**DOI:** 10.5334/joc.319

**Published:** 2023-09-04

**Authors:** Yi Feng, Anja Pahor, Aaron R. Seitz, Dennis L. Barbour, Susanne M. Jaeggi

**Affiliations:** 1 University of California, Irvine, School of Education, School of Social Sciences (Department of Cognitive Sciences), Irvine, California, USA; 2 University of California, Riverside, Department of Psychology, Riverside, California, USA; 3 Northeastern University, Department of Psychology, Boston, Massachusetts, USA; 4 University of Maribor, Department of Psychology, Maribor, Slovenia; 5 Washington University in St. Louis, Department of Biomedical Engineering, St. Louis, Missouri, USA

**Keywords:** Machine learning, Individual differences, Working memory

## Abstract

People differ considerably in the extent to which they benefit from working memory (WM) training. Although there is increasing research focusing on individual differences associated with WM training outcomes, we still lack an understanding of which specific individual differences, and in what combination, contribute to inter-individual variations in training trajectories. In the current study, 568 undergraduates completed one of several N-back intervention variants over the course of two weeks. Participants’ training trajectories were clustered into three distinct training patterns (high performers, intermediate performers, and low performers). We applied machine-learning algorithms to train a binary tree model to predict individuals’ training patterns relying on several individual difference variables that have been identified as relevant in previous literature. These individual difference variables included pre-existing cognitive abilities, personality characteristics, motivational factors, video game experience, health status, bilingualism, and socioeconomic status. We found that our classification model showed good predictive power in distinguishing between high performers and relatively lower performers. Furthermore, we found that openness and pre-existing WM capacity to be the two most important factors in distinguishing between high and low performers. However, among low performers, openness and video game background were the most significant predictors of their learning persistence. In conclusion, it is possible to predict individual training performance using participant characteristics before training, which could inform the development of personalized interventions.

## Introduction

If you happen to have ever been on an athletic team, you probably noticed that even when team members are given the same training program, their progress varies greatly. Some athletes respond well and show huge improvements at the beginning of the training program. Others improve very slowly, even if they practice the same amount of time as those who show rapid improvements. It is undeniable that there is considerable variation between people during training and the extent to which they learn and improve their performance. The question remains, however, as to what factors are responsible for these inter-individual variations in learning adaptation. In this study, we explore the variability in learning to answer this question. We use working memory (WM) training as a proxy for learning since WM – the cognitive process that facilitates temporarily holding and manipulating information for a short period of time – is a good indicator of general skill learning ability (Cowan, 2008).

### Heterogeneity of Learning Trajectories

There is an ongoing debate around the universal effectiveness of WM training (Au et al., 2016; Jaeggi et al., 2008, 2011; Klingberg, 2010; Melby-Lervåg & Hulme, 2016; Pahor, Seitz, & Jaeggi, 2022; Thompson et al., 2013; Von Bastian et al., 2013), and it has been argued that a major source of variability can be attributed to individual differences (Borella et al., 2017; Jaeggi et al., 2014; Traut, Guild, & Munakata, 2021). This heterogeneity in WM training is evident in both training outcomes (Katz et al., 2021; Meiran et al., 2019; Redick, 2019; Wiemers et al., 2019), as well as in learning trajectories that emerge during the intervention process (Flak et al., 2019; Holmes et al., 2009; Karbach et al., 2015). Additionally, Ackerman (2007) demonstrated that practicing a complex task accentuates existing individual differences, implying that the training process itself plays a role in shaping these variations. Notably, learning trajectories vary significantly between individuals, even when performing the same task (Bürki et al., 2014; Guye et al., 2017; Wiemers et al., 2019), and have shown to predict learning outcomes (Karbach, Strobach, & Schubert, 2015; Jaeggi et al., 2011), however, individual differences in learning trajectories within the cognitive training literature remain relatively sparse (e.g., Bürki et al., 2014; Guye et al, 2017; Karbach et al., 2017; Matysiak, Kroemeke, & Brzezicka, 2019, Ørskov et al., 2021).

Könen and Karbach (2015) emphasized the need for research that includes comprehensive training trajectory data to better understand the factors contributing to individual differences in skill learning. They suggested that analyzing time-series training data can provide valuable insights, such as identifying sessions where participants improved, their peak performance, the stability or variation between sessions, and the turning points when stability changed. Accordingly, these findings will provide insights on how to design customized training settings for individuals based on their learning curves. However, only a few studies have examined individual differences using dynamic training data (Bürki et al., 2014; Guye et al., 2017; Karbach et al., 2017). These studies have typically employed latent growth models to analyze training trajectories, assuming linear or quadratic changes in performance. However, these models have limitations, as they assume predefined trajectories applicable to all participants, and stable performance changes throughout the intervention period. Thus, methods to capture individual learning trajectories beyond these indicators are needed, for example by including when and how stability has changed, which is the goal of the present paper.

### Factors Affecting Skill Learning

Several factors have been identified as determinants of skill learning. These factors encompass both pre-existing cognitive ability and non-cognitive traits such as interests, personality, motivation, and self-efficacy, all of which have shown to influence skill acquisition (Ackerman, 2007; Von Bastian & Oberauer, 2014; Katz et al., 2021). However, no specific theory has unified all these findings so far. Bandura’s social-cognitive framework, which considers interactions between behavioral, person-centered, and environmental factors, offers a comprehensive perspective on individual learning and performance (Bandura, 1986). In this study, we rely on Bandura’s framework and focus on prominent factors that have shown to impact learning outcomes in previous studies within the WM training literature, categorizing them into three groups: baseline cognitive ability, person-related characteristics, as well as environmental and experience-related factors.

Baseline cognitive ability, which refers to pre-existing ability before any intervention, significantly influences skill learning (Bürki et al., 2014; Foster et al., 2017; Guye et al., 2017; Karbach et al., 2017; Zinke et al., 2012, 2014; Ørskov et al., 2021). Various measures of baseline cognitive ability have been found to impact skill learning outcomes, which include general cognitive skills (such as fluid reasoning), as well as specific tasks that share processes with trained tasks. However, there are still uncertainties regarding the relationship between baseline cognitive ability and learning performance. Some studies suggest that individuals with the highest initial performance on trained tasks show the most improvement during WM training (magnification account or Matthew’s effect; “rich-get-richer”; e.g., Bürki et al., 2014; Foster et al., 2017; Guye et al., 2017; Ørskov et al., 2021). Conversely, other research indicates that individuals with the lowest initial training performance experience the largest gains in WM training (compensation account; “catch up”; e.g., Jaeggi et al., 2011; Karbach et al., 2017; Zinke et al., 2012, 2014). Additionally, learning outcomes might be differentially impacted by general cognitive ability or specific skills (Karbach et al., 2017; Wiemers et al., 2019). To shed more light on this issue, our study aims to examine the impact of baseline performance by using general cognitive ability (measured by fluid reasoning), composite WM skills, as well as specific tasks sharing processes with trained tasks as predictors, in order to determine their impact on training results.

Person-related characteristics, including motivational factors, such as growth mindset or self-perceived cognitive failures, as well as certain personality traits have also shown to influence skill learning (Guye et al., 2017; Katz et al., 2021; Ørskov et al., 2021). For example, individuals with growth mindsets view training as a means to enhance their abilities, leading to increased engagement and improved performance (Dweck, 2000). Moreover, those reporting more cognitive failures have shown to be more likely to participate in a WM intervention and persist with them (Jaeggi et al., 2014). Personality traits like conscientiousness and neuroticism have also been linked to cognitive and emotional regulation, aiding individuals in maintaining diligence and managing negative emotions during training (see review Katz et al., 2021). Other personality traits may also play a role in skill learning. For example, individuals who show high grit and ambition tend to persevere despite setbacks and hold high expectations for their performance, leading to increased engagement (Datu, 2021; Duckworth et al., 2007). However, the relationship between these person-related characteristics and learning outcomes has been inconsistent (Guye et al., 2017; Nemmi et al., 2016; Thompson et al., 2013). The scarcity of studies incorporating these person-related factors and the small sample sizes used in most studies present further challenges, which we aim to address in our study.

Environmental and experience-related factors also contribute to skill learning (Sigmundsson et al., 2017). Maintaining good physical health is crucial for optimal brain function and learning across the lifespan (Khan & Hillman, 2014; Macpherson et al., 2017). Although the direct impact of health on skill learning is not extensively studied, several studies have shown that physical exercise benefits spatial memory, working memory, and executive attention (see review Cassilhas et al., 2016). Furthermore, prior experience and exposure to cognitive training regimens or computerized tasks can promote skill learning (Berard et al., 2015; Steyvers & Schafer, 2020). Socioeconomic status (SES) has shown to be an indicator of individuals’ exposure to experiences relevant to cognitive training including WM training, such as cognitive tutoring, but also playing computer games or educational puzzles (Duncan & Magnuson, 2012). Moreover, experience with computers and playing video games has been found to predict performance in cognitive training games, suggesting that gaming experience can enhance perceptual processing and learning more generally (Steyvers & Schafer, 2020; Zhang et al., 2021). This growing literature highlights the potential importance of SES and video game experience in learning outcomes, but it warrants further exploration.

### Predicting Trajectories

Previous research has examined individual difference variables to predict learning trajectories separately. However, skill learning is influenced by a combination of factors, and these factors are often interrelated, and the relationship might not be linear (e.g. Guye et al., 2017). Moreover, earlier studies have used limited approaches to examine links between individual difference factors and training performance. Even when using multiple regression or latent growth models, the assumed relationship between factors and training performance remains linear (Bürki et al., 2014; Guye et al, 2017; Karbach et al., 2017), which inherently restricts the interpretation of these findings. Even structural equation modeling, which uncovers complex relationships among variables, primarily relies on linear relationships and assumes independence among moderators. Research has revealed that when analyzing the impact of personality on cognitive ability through regression analysis, quadratic associations account for a significant amount of variance beyond the effects of linear factors (Major, Johnson & Deary, 2014). Thus, to comprehensively understand the relationship between individual difference factors and learning performance, it is necessary to employ an approach that can combine several factors and enable correlations between factors without assuming a linear relationship with training performance. Furthermore, in order to better understand underlying mechanisms of learning trajectories, focusing on the predictive capabilities of individual difference factors could help uncover the intricate and complex relationships between multiple factors and learning outcomes and forecast future behavior (Luan & Tsai, 2021; Orrù et al., 2020; Yarkoni & Westfall, 2017).

Machine learning algorithms serve as the most advanced tools for prediction, as they can handle numerous predictors and their intricate relationships (Yarkoni & Westfall, 2017). Unlike traditional statistical explanatory methods that rely on assumptions about variable relationships, many machine learning algorithms employ nonlinear algorithms and do not make such assumptions. Specifically, the fundamental principle behind machine learning models is that they learn the relationships between variables in the provided data, enabling the prediction of future behaviors of new samples (Mohri et al., 2018).

In this study, we utilize machine learning models to predict learning trajectories in a WM intervention conducted by more than 500 participants (a large sample size in the cognitive training field). To do so, we rely on a set of predictors that have been identified as relevant in previous literature including baseline cognitive abilities, person-related characteristics, and environmental factors. We hypothesize that individuals’ learning trajectories can be categorized into different patterns, and that a combination of individual difference variables can successfully predict these patterns using machine learning models. Given that many studies emphasize the role of baseline cognitive abilities in influencing training gains, we hypothesize that this factor will be the most powerful predictor.

## Methods

### Participants

Participants were recruited from the Universities of California, Irvine and Riverside. In total, 568 undergraduates from diverse socioeconomic backgrounds completed the WM intervention as well as several surveys and assessments. The data are combined from three studies. Study 1 was conducted from 2017 to 2018 (266 participants); Study 2 from May to August 2020 (138 participants); Study 3 from May 2020 to December 2021 (164 participants). Study 1 was a pre-covid in-lab study, while Studies 2 and 3 were administered online due to the pandemic. Participants’ age ranged between 18–57^1^ (*M_age_* = 20.37, *SD* = 3.63), and 64% of them were women. They were diverse in terms of ethnicity and race, with 33% identifying as Hispanic or Latino, 3% identifying as African American, 46% Asian, 14% Caucasian, 0.7% American Indian, 8% Biracial/Mixed race, 0.9% Native Hawaiian or Other Pacific Islander, and 24% reporting others. The study procedures were approved by Institutional Review Boards at both sites, and each participant received $80 or $120 as financial compensation for the online or in-lab studies, respectively.

### Training program

An app-based N-back training program was developed in-house by University of California Riverside Brain Game Center (“Recollect the Study”; available on Google Play and Apple App Store; cf. also https://apps.apple.com/us/app/recollect-the-study/id1217528682; a video can be found here: https://www.youtube.com/watch?v=zhgL8Oe42Yk). In the game, participants were presented with a series of stimuli consisting of different shapes and colors (see supplementary Figure S1). They were asked to respond to stimuli that matched the stimulus presented N items back. For instance, in a 1-back task, participants respond if the current stimulus is the same as the stimulus presented 1 item before. The task difficulty was adaptively adjusted based on participants’ performance, with higher levels of N increasing the task difficulty. Participants need to keep track of more items with higher N levels. With the same task requirement, we employed different training environments, including gamified and non-gamified variants, along with 10 adaptive algorithms to adjust the N-level progression (cf. Sandeep et al., 2020). For example, one algorithm required 3 hits to level up and 2 errors to level down within a block, while other algorithms utilized different conditions, such as 3 hits to level up and 3 errors to level down. For the purpose of the present work, our objective is to identify the influential factors that contribute to learning trajectories, irrespective of the specific training conditions. Therefore, we did not include the training condition as a predictor variable. Still, to explore whether training conditions would contribute variance to training trajectory, we added these variables to our model as described in the supplementary materials. Notably, the model performance did not change substantially (Table S7).

Each participant completed one 40-minute training session per day, with a break after 20 minutes, for a total of 10 sessions. Each session consisted of multiple blocks, and each block lasted 2–3 minutes that included 20 plus N trials. The dependent variable was the weighted average N level achieved during a session. Specifically, each N-level was multiplied by the number of trials associated with that block, then divided by the total number of trials. It represents the average level that participants achieved in a particular session (cf. Pahor et al., 2022).

### Predictor variables

Before training, participants completed several cognitive assessments and a series of self-report questionnaires capturing demographic information including SES and video game playing experience, personality traits and other characteristics like growth mindset.^2^ All assessments and questionnaires were conducted on tablets and/or via Qualtrics software. The descriptive information for all predictors is provided in supplementary materials Table S1.

#### Cognitive Assessments

##### WM updating

We employed an untrained variant of the N-back task to measure participants’ updating skill, which served as a proxy of specific training task baseline measure, using either pictures of animals or vehicles (Pahor et al., 2022). All participants completed 1-back, 2-back, and 3-back levels (in that order). Each N-level consisted of 30 trials with nine targets. Each stimulus was presented 2.5 seconds with a 500 ms interval. Accuracy for each N-level was calculated as hits/(hits+misses+false alarms). We averaged z scores of 2-back and 3-back accuracy as the predictor variable.

##### WM capacity

To examine WM ability in different contexts, we used two WM tasks. **Letter-number task (LN)** was a verbal WM task similar to the letter-number sequencing subtest of the Wechsler Adult Intelligence Scale-III (Wechsler, 1997). Participants were required to remember and sort a mixed order of letters and numbers. For instance, the sequence ‘H8T3K5’ would be sorted into ‘358’ and ‘HKT’. Set size was the length of the sequence, and the task started from set size 2 and ranged to 15. Two trials were presented per set size. The task ended if both trials on the same set size were recalled incorrectly. The outcome variable was the highest set size achieved where at least one trial was correct. **Corsi Blocks Forward (CF)** was a visuospatial WM task inspired by traditional e-Corsi Block tests (cf. Ramani et al., 2020). In the task, participants saw a sequence of gophers appear in different holes; after that, participants were asked to tap on them in the correct order. Each gopher was displayed for 1.5 seconds and the interstimulus interval was 0.5 seconds. Set size ranged from 2 to 10, and the task began with the lowest set size. Similar to the letter-number task, there were two trials per set size, and the game ended when both trials were recalled incorrectly. The outcome variable was the highest set size achieved. We used the average z score of both tasks (LN and CF) to reflect WM baseline capacity.

##### Inhibitory control

We tested inhibitory control (IC) as a predictor given extant research suggesting that IC is highly correlated with WM (Robert et al., 2009), however its role in WM skill learning is to date unknown. We used a countermanding task, which is a modified version of the Simon and spatial Stroop task, measuring the interference caused by spatial incongruent stimuli (Davidson et al., 2006; Diamond, 2013; Wright & Diamond, 2014). In this task, participants were presented with visual stimuli in the form of pictures of dogs and monkeys appearing on either the left or right side of the screen. They were instructed to tap on one of two green buttons in response to these stimuli (Ramani et al., 2020). For dogs, participants were required to tap on the button that was on the same side of the screen (congruent trials); however, when the participants saw a monkey, they were required to tap on the button that was on the opposite side (incongruent trials). The outcome measure was the reverse z score of the average response times spent on incongruent trials minus the time on congruent trials, thus a higher z score reflecting higher IC ability.

##### Fluid reasoning

We used the University of California Matrix Reasoning Task (UCMRT) to capture fluid reasoning skills (Pahor et al., 2019). Participants were shown a 3×3 matrix of stimuli with a missing element. They were required to select the missing part from eight answer alternatives to complete the pattern. There were 23 problems with a time limit of 10 minutes. The predictor variable was the proportion of correct answers.

#### Person-Related Factors – Self-Report Measures

##### Growth mindset

We used the Growth Mindset Scale (Dweck, 1999) to assess participants’ beliefs about the malleability of intelligence. For our study, we replaced the word “intelligence” with “cognitive ability”, e.g. “No matter who you are, you can significantly change your cognitive ability”. In Study 1, participants responded on a 6-point scale from “Strongly disagree” to “Strongly agree”. However, in Study 2 and 3, the “Strongly disagree” option was not displayed by mistake. Thus, we only included data from study 1 and calculated the sum of 5 items as the predictor variable.

##### Cognitive failures

To assess self-reported failures in memory, we selected 8 items from Broadbent et al.’s cognitive failure questionnaire (CFQ) (1982) as modified by McVay and Kane (2009). Participants were asked to report how frequently they encountered each scenario in the last twelve months (e.g. “Do you forget to give a message to somebody as you were requested to do?”) on a Likert scale from 1 (Never) to 5 (Very often). Sum of all items was used as a predictor variable.

##### Grit and Ambition

Grit is described as a positive trait toward passion and perseverance. There were 8 statements to assess grit (Duckworth & Quinn, 2009), e.g. “I finish whatever I begin”. Participants evaluated each statement with 5 points from “Very much like me” to “Not like me at all”. Ambition is a concept showing the desire for success and captured by 5 items (Duckworth et al., 2007), like “I am a hard worker” using a 5-point scale as Grit. Since ambition is commonly related to grit (*r* = .518 in our dataset), we used a composite score averaging across the two scales.

##### Personality Traits

We used the 40-item Mini-Markers questionnaires (Saucier, 1994) to capture participants’ big five personality traits (Neuroticism, Extraversion, Openness, Agreeableness, Conscientiousness). Each personality dimension was represented by 8 items consisting of adjectives. Participants were asked to rate how true the items are about themselves using a 5-point likert scale. Each trait was represented by its average score across corresponding items, serving as a predictor variable.

#### Environmental Factors and Experience

##### Socio-economic Status (SES)

SES was inferred by both participants’ self-reported subjective SES and their parents’ education level. We used the MacArthur Scale of Subjective Social Status–Youth Version (Goodman et al., 2001) in which participants were shown two 10-step ladder pictures representing their community and the whole country. Then, they were asked to rate where their families would be on ladders. From the top of the ladder to the bottom were coded from 10 (the highest SES) to 1 (the lowest SES). The overall score of the two ladders reflected their self-reported SES level. In our dataset, the overall score ranged from 4 to 20 (average level = 11). Parents’ education level (less than high school, high school diploma or equivalency, associate degree (junior college), Bachelor’s degree in college, Master’s degree, Doctoral degree, professional degree (JD, MD), and other specifics) was also collected and coded from 0 to 6. The mean score for parents’ total education ranged from 1 to 7 (average score = 4.75) in our dataset, indicating a wide range of SES. Both parents’ education level along with self-reported SES level were used as predictors.

##### Video Game Background

We assessed participants’ gaming experience using a video game questionnaire (VGQ) as used in previous work (Waris et al., 2019). In our survey, participants estimated how many hours they spent in 6 categories of video games (Action with shooters like Call of Duty, Action with adventure like Grand Theft Auto, Non-action role playing game like World of Warcraft, Real-time strategy like Starcraft, Turn-based strategy like Civilization, Music games like Guitar Hero) per week in the past year. Their estimations were done using a 6-point scale that included Never, 0 to 1 hour, 1 to 3 hours, 3 to 5 hours, 5 to 10 hours, 10+ hours. Consequently, we converted the individual sum of video game hours within the populations of Study 2 and Study 3 into z-scores. We also calculated z-scores within Study 1. Finally, we merged the z-scores obtained from all three studies.

##### Health Status

A total of 3 self-rated items were used to assess participants’ satisfaction with their health (e.g. How satisfied are you with your present physical health/psychological health/physical fitness?). Each item is assessed by a 5-point scale, from 1: Very dissatisfied to 5: Very satisfied. The raw scores served as 3 predictors.

##### Bilingualism

We created a dummy variable to indicate whether or not participants were bilingual (0 = monolingual, 1 = bilingual). 80% of our sample identified as bilingual (N = 403).

### Analytic plan

**Figure 1:**
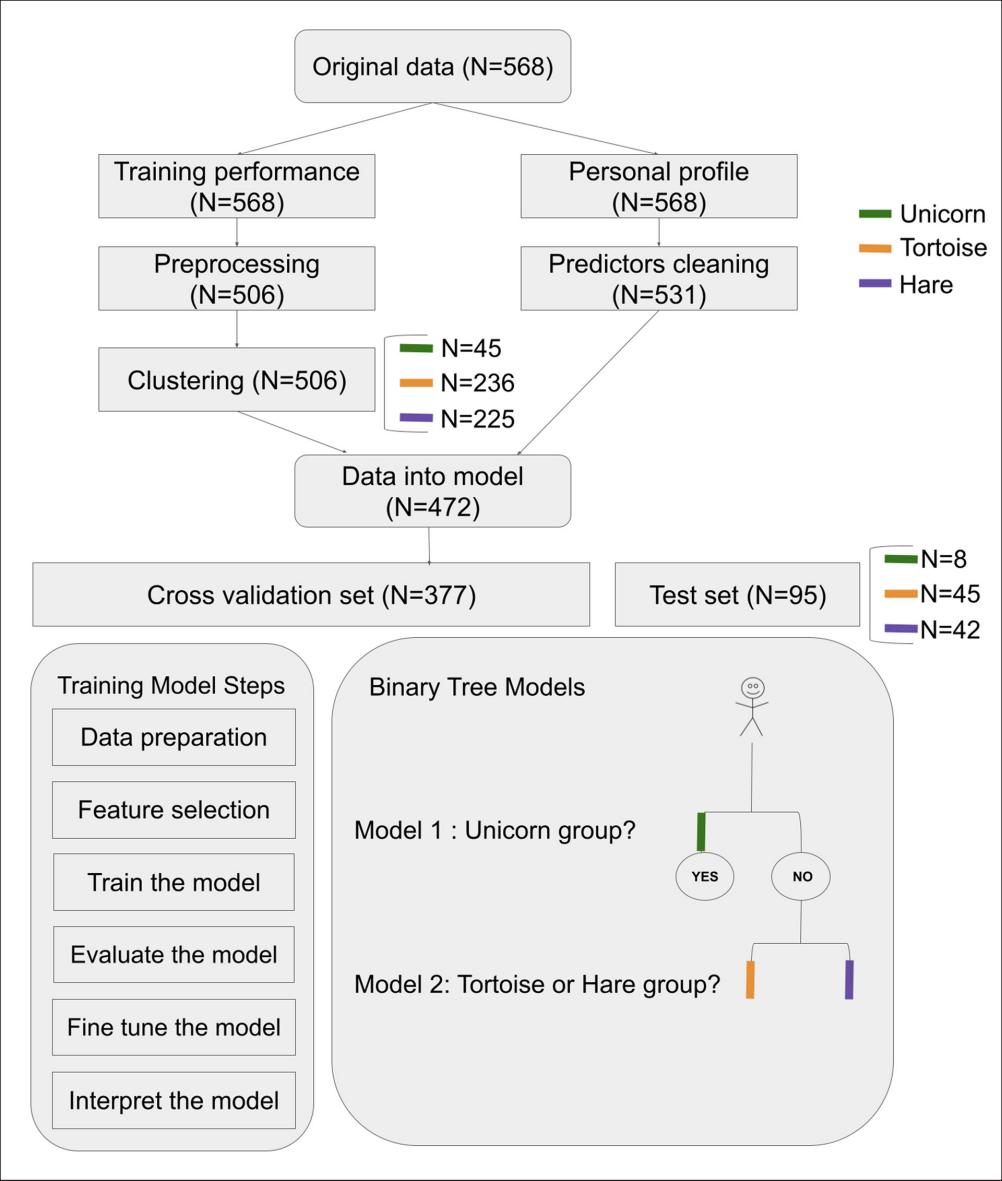
General Flowchart of Analytic Plan. *Note*: Three colors represent different groups identified by clustering.

The data analysis comprised two primary steps: preprocessing and training the machine learning models. Preprocessing involved handling missing values and outliers separately in the training data and predictors. Following that, participants were grouped based on the training data, and the machine learning models were trained to predict their respective groups (refer to Figure 1 for the flowchart).

#### Training performance preprocessing

To identify outliers during training, we calculated each participant’s average n-back level and standard deviation (SD) across training sessions. Since the distribution of SD and average performance were skewed (Skewness = 8.28 and 7.77, respectively), outliers were identified by a skewness-adjusted boxplot tool (Hubert & Veeken, 2008). Afterward, we excluded 20 outliers whose average performance or SD did not fall within the skewness-adjusted .25 to .75 quantile range (mean of outliers’ average n-back level = 16.25, SD = 7.50). After deleting outliers, we applied piecewise linear models to fit each participants’ training trajectory. We divided the training curve of each participant into two parts, each part was fitted with a linear curve, and the two straight lines were connected at a knot (Ørskov et al., 2021). We restricted the position of the knot not to appear in the first or last session. We were not able to fit 42 participants’ training data with the piecewise linear model because their performance did not change across all sessions, i.e., they essentially did not show any learning. Further analysis excluded 11 of them since they had only been exposed to the 2-back task throughout the entire training period due to a specific algorithm setting. The remaining 31 participants who kept flat during training sessions (i.e. starfish group) were treated as a specific group discussed in supplementary materials. For each of the remaining 506 participants, we used a data-driven approach to put the knot at different time points (i.e. sessions) and determined the optimal knot location by the best model fit (largest r square). With the optimally fitted knots, the mean r square across all participants was .73. We also used linear function, quadratic function, exponential function, and logarithmic linear function to fit participants’ training trajectories, however, the average model fit was not as good as the one using piecewise linear models (average r square = .47, .62, .44, and .52, respectively). After fitting training curves with piecewise linear models, we observed that the knot locations of the curves varied among participants (Mean knot = 5.13, Skewness = .18). Additionally, the slopes of the first part (slope1) also showed variation among participants (Mean slope 1 = .45, Skewness = 2.55). In contrast, the slopes of the second part (slope2) were centered around 0 (Mean slope 2 = .00, Skewness = 5.43). The informative variation in the population seems to be concentrated in the knot and slope 1. For this reason, we used participant’s knot location and only slope1 to represent training trajectory.

We decided to identify different learning patterns using these two indicators (i.e., knot location, slope1) instead of directly utilizing the continuous indicator values. This decision was made due to the reason that when we attempted to use slope1 as the target and trained regression models (excluding the no change group), the prediction performance yielded RMSEs above 0.35 (see supplementary Table S4). This outcome suggests that our predictors might not have sufficient sensitivity to accurately distinguish subtle differences in learning rates. Therefore, we employed clustering to identify learning patterns from both the participants’ knot location and slope1, and treated participants who did not exhibit any changes during training as a single learning pattern.

#### Clustering

**Figure 2:**
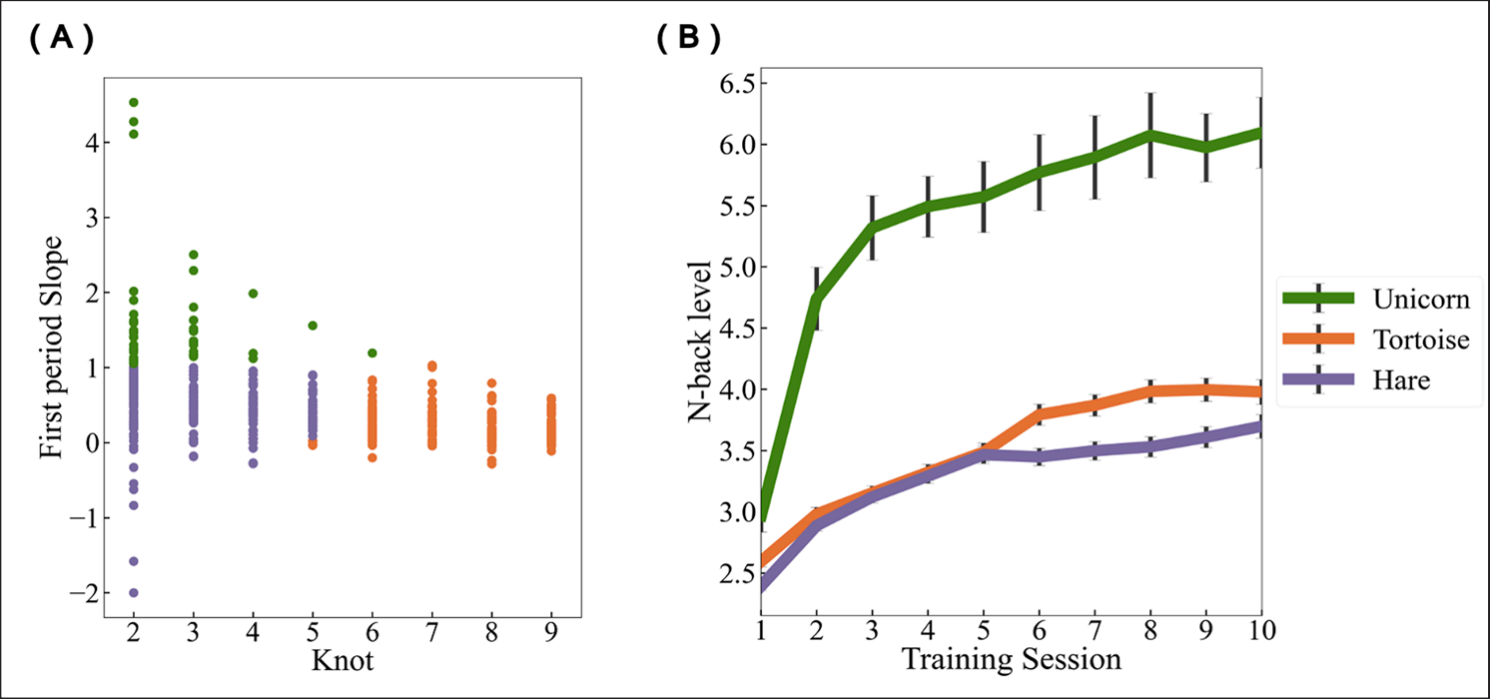
Learning Patterns Identified by Clustering. *Note*: A) Scatter plot of participant’s optimal knot location (x axis) and initial training gain (slope1; y axis) as a function of the three learning clusters. B) Average learning trajectories of the three clusters across the 10 training sessions. Error bars represent ±1 standard error of the mean.

Clustering is an unconstrained method that does not make any assumption of the performance change curve and helps to determine the best grouping among participants. To detect different training patterns underlying participants’ training trajectories, we used the K-means algorithm, which is commonly used in clustering (Likas et al., 2003). The K-means algorithm is intended to partition data into k clusters so that data points (participants in our case) within the same cluster are more similar, and points in different clusters are more dissimilar. Similarity between two participants was determined by the Euclidean distance between them, specifically, calculated by their knot location and slope1. K-means tries to minimize distances within a cluster and maximize the distance between different clusters. We implemented K-means to divide participants into three clusters based on their knot location and slope1 across training (both standardized). To measure the goodness of the clustering result, the average silhouette score was computed. Silhouette scores are used to measure how dense and well-separated the clusters are. Silhouette scores range from -1 to 1, with higher scores indicating that the clusters are clearly distinguishable. The silhouette score of the three classes in our dataset was .47. We tested alternative clustering into two or four clusters (Silhouette score = .49 and .45, respectively) or more clusters (see supplementary Figure S2), and even though the silhouette score using two clusters was numerically higher than the three-cluster solution, we decided to work with the three clusters given that the two-cluster solution would likely fail to capture more subtle training patterns. Afterwards, participants were divided into three groups as determined through clustering, consisting of 45, 236, and 225 participants, respectively (see Figure 1). For the remainder of the paper, we refer to the participants in those three groups as “Unicorns”, “Tortoises”, and “Hares”, which captures their training trajectories (Figure 2).

#### Predictors cleaning

Since many machine learning algorithms do not support missing values, we addressed missing values on predictor variables first. First, we checked the missing rate for each predictor. Growth mindset was excluded because it had about 61% missing values due to the aforementioned errors with administration during study 2 and study 3. Missing rates of other predictors ranged from 0 to 13.7%. Second, we looked at the missing rate for each participant. We conducted a Little’s Missing Completely at Random (MCAR) test, which resulted in a non-significant value of .778. This suggests that the missing pattern in our data was completely random. Nonetheless, the proportion of missing data is directly related to the quality of statistical inferences (Dong and Peng, 2013). To omit obvious loss of information, we decided to exclude participants who did not complete 50% of the survey with above nine missing values out of 18 predictor variables. The remaining missing values were imputed using Multiple Imputation by Chained Equations (MICE), a method in which each feature with missing values is modeled as a function of its neighboring features in a round-robin fashion (Zhang, 2016). Specifically, MICE was implemented using the multivariate imputer with the Bayesian Ridge regression model. The imputer followed an ascending imputation order and considered all features. The proportion of imputations (number of imputed cases/number of total cases) is provided in Table S1. Afterwards, we combined the cleaned predictor variables of the participants with their clustering results. Since we did data cleaning for training performance and predictors in parallel, we needed overlapping cleaned cases that do not contain outliers or excessive missing data in both intervention and predictor variables. In total, there were 472 cases left for classification.

#### Classification preprocessing

Classification preprocessing is the first step in the application of machine learning algorithms (Albon, 2018). The objective is to increase the signal-to-noise ratio and eliminate redundant or irrelevant information. Our preprocessing included Train-test split, Standardization, Oversampling, and Feature selection. Train-test split is used to estimate the performance of machine learning algorithms when they are used to make predictions on data not used to train the model. We used a stratified shuffle split method randomly picking 20% participants as a test set having equally balanced classes. The remaining 80% of participants were used as a training set to feed classification models. All predictors in both train and test sets were standardized separately to keep them in the same range and treated the test set as a completely new dataset. Specifically, the train set was standardized within the set, while the test set z score was calculated based on the mean and standard deviation from the train set: (value from test set – corresponding mean from train set)/(corresponding standard deviation from train set). By doing so, we utilize information from the train set to better scale the test set due to the limited sample size of the test set. We used oversampling and feature selection as strategies given that we were dealing with a relatively small data set for machine learning field. Oversampling was used to prevent the reduction of the model’s predictive power caused by an unbalanced data set. In this case, we used the SMOTENC (Synthetic Minority Over-sampling Technique for Nominal and Continuous). SMOTENC creates new synthesizing predictors’ data for the minority class, based on those that already exist (Chawla et al., 2002). It can handle dataset containing numerical and categorical features. We used oversampling to increase the sample size of the minority group to be the same as the majority group in the train set. Feature selection allows us to remove unneeded features that do not contribute to prediction and to avoid overfitting (Guyon & Elisseeff, 2003). Specifically, we used a robust feature selection method on the train set: L1 regularization or so called LASSO (Least Absolute Shrinkage and Selection Operator) (Tibshirani, 1996). With the LASSO method, the regression coefficients are reduced to zero, which regularizes the model parameters and avoids overfitting. Features with non-zero coefficients will be kept in training models. To choose the best regularization parameter C (inverse of regularization strength), we explored values between 0 and 1, with the best C set to be 0.1. To determine the effectiveness of feature selection, we compared the model performance with all features included. The results can be found in the supplemental materials, Table S5.

#### Binary tree model

The clustering procedure assigned each participant a label, which was the target we needed to predict with the machine learning model. This known target prediction process required the use of supervised classification learning models. Specifically, we used a hierarchical binary tree model to divide multiclass classification into two binary classification models (see Figure 1). We chose a binary tree model over a multiclass model due to the varying performance observed between the labels. While we initially attempted a multiclass model, it did not yield satisfactory results, likely due to the significant differences in distinction between the unicorn and non-unicorn group, as opposed to the distinction between the tortoise and hare group. Hence, we decided to employ two separate algorithms to effectively differentiate these label groups: The target of model 1 was to detect unicorn group, whereas the target of model 2 was to distinguish between the tortoise and hare group. The two models were trained separately with the same classification preprocessing approach. For each model, after feature selection, we first applied a variety of algorithms with default parameters to train the data in order to avoid personal bias in choosing a classification model (Géron, 2019). This was achieved via a model selection function in the scikit-learn library (version 1.1.2) in Python (Pedregosa et al., 2011), which evaluates several algorithms’ performance (e.g., Logistic Regression, Decision Tree, etc, cf., supplementary materials; Table S6). The evaluation approaches were discussed in the next paragraph. Model 1 and model 2 were selected based on different criteria. For Model 1, our primary focus was on accurately detecting unicorns and avoiding the omission of positive instances. On the other hand, Model 2 aimed to differentiate between hares and tortoises with utmost accuracy. We found the Radial Basis Function (RBF) Kernel Support Vector Classifier (model 1) and Random Forest algorithm (model 2) to be the most promising models. RBF kernel support vector classifier is a method that is responsible for finding a non-linear decision boundary to separate different classes and maximize the distances between cases at boundaries (Schölkopf et al., 2004). It can handle nonlinear data with high dimensions. Random Forest is an ensemble model based on a number of Decision Tree classifiers (DT). Each DT will learn from data and make a flow of if-then-else rules to predict new instance labels. RF then averages all trees’ decisions and makes a final prediction. Random Forest is also a commonly used approach in precision education using machine learning (Luan & Tsai, 2021). Random Forest has advantages in dealing with unbalanced data and avoiding overfitting by restricting how many if-then-else rules in each DT. Random Forest has no assumption of data structure that can capture nonlinear relationships and allow interactions among predictors (Denisko and Hoffman, 2018).

After model selection, we applied an exhaustive search over specified parameter values for two models and selected the optimal parameters according to the model performance. When evaluating the performance of the model, we used the cross-validation method, specifically the 10-fold method. Ten-fold cross-validation is an estimate of the generalization performance of a model trained on 9/10 samples (Berrar, 2018). Unlike the traditional division of data into a training and testing set, 10-fold cross-validation treats each fold as a testing set, and the remaining participants as the training set. In that regard, we trained and validated a model 10 times and looked at their average performance. In addition, we evaluated model performance with widely acceptable performance metrics including A) a confusion matrix, which indicates the combination of true labels and predicted labels; B) The mean accuracy across all 10 folds, evaluated using the f1_weighted scoring metric that considers the performance across all classes; C) F1 score as a weighted harmonic mean of precision and recall calculated based on confusion matrix (for the formula see supplementary materials), with scores ranging between 0 and 1 (scores closer to 1 indicate better classifiers). Due to the hierarchical binary tree model we used here, the chance level for prediction is .50 for each binary model within it. When we apply this binary tree as a whole, the chance level for predicting membership in any of the three groups is approximately .33. It is worth noting that among our training data, some of them were created by the oversampling method (~50% for model 1). This part of the data was just to balance each class to help train better models. Therefore, when validating the model, we eliminated those synthetic data so that all the model performance indicators only involve the original dataset.

#### Model interpretation

After training the machine learning model, we applied a method called SHAP (SHapley Additive exPlanations) to determine the extent to which each feature contributes to making predictions in final models (Lundberg and Lee, 2017). This method can demonstrate feature importance, both globally and locally, to supervised learning of labeled data. The SHAP are based on concepts from cooperative game theory, which assigns each feature an importance value based on whether the feature is present or not during SHAP estimation (Lundberg and Lee, 2017; Lundberg, Erion & Lee, 2018). Specifically, for each case, the final model provided the predicted probability of belonging to a specific label. The difference between this probability and the baseline probability (the label’s proportion in the population) was quantified by SHAP values, indicating the predictors’ contribution. A positive SHAP value indicates a positive contribution, meaning it tends to predict the label, while a negative value indicates a tendency to not predict the label. The important predictors are those with higher average absolute SHAP values across all cases. Since different explanation formulas are suitable for different types of models, we utilized a kernel-based explainer for the RBF Kernel Support Vector Classifier (model 1), and a tree-based explainer for the Random Forest Classifier (model 2).

## Results

### Learning pattern

As shown in Figure 2, the three colored curves represent the average training trajectories of the three learning patterns as determined by the K-means method. Unicorns outperform both other groups, showing a much higher performance from the beginning and extending their high performance throughout training; Tortoises maintain a regular rate of daily progress compared to hares, which reach an early asymptote. Mean training curves for each group are shown in the supplementary materials (Figure S3).

The results of subsequent ANOVA analyses revealed that participants started at different n-back levels during session 1 with significant differences emerging between groups at the outset (*F_2,503_* = *16.98, p < .001*). Bonferroni post hoc tests confirmed that the unicorn group (Mean WM span = 2.97) significantly outperformed the tortoise (Mean WM span = 2.59) with *p < .001, Cohen’s d* = *0.53* and hare groups (Mean WM span = 2.39) with *p < .001, Cohen’s d* = 0.94. The unicorn group also showed a high growth rate (Mean slope 1 = 1.63) over the first three sessions (Mean knot location = 2.62), and kept its high performance (Mean slope 2 = 0.07). The tortoise and hare groups showed slower growth rates (Mean slope 1 = .22 and .45, respectively), and tortoises progressed significantly more slowly than hares (*Cohen’s d* = *0.77*). The tortoise group, however, continued to improve for a longer period of time than the hare group (Mean knot = 7.42 versus 3.23, *Cohen’s d* = *3.52*). Finally, the tortoise group performed slightly better than the hare group at the end of training (performance at session 10: 3.98 versus 3.70, *Cohen’s d* = *0.19, p* = *.047*).

### Model performance

With this model, we applied the feature selection method described above to determine the optimal subset of predictors. WM, Fluid reasoning, Inhibitory control, Grit and Ambition, Consciousness, Openness, Extraversion, Neuroticism, SES, Video game background, Health status, and Bilingualism were included in the final binary model 1; Inhibitory control, Cognitive failure, Openness, Neuroticism, Extraversion, and Video game background were included in the final binary model 2.

As shown in Table 1, two models performed well on the cross validation set. Model 1 (RBF Support Vector Classifier), when identifying unicorn participants, achieved an average accuracy of .90 across 10 folds validation sets (*SD* = .03), with an F1 score of .46. Model 2 (Random Forest Classifier) showed less power in predicting whether participants were from the tortoise or hare groups. The average accuracy of .64 across 10 folds (*SD* = .05), with an F1 score of .65.

**Table 1:** Confusion Matrix of Binary Tree Model Performance on Cross Validation Set.


	MODEL1 (N = 377)	PREDICTED CLASS	

		UNICORN	NON-UNICORN

True class	Unicorn	**28 (.88)**	4 (.12)

	Non-Unicorn	62 (.24)	**283 (.82)**

	**MODEL2 (N = 345)**	**TORTOISE**	**HARE**

True class	Tortoise	**114 (.64)**	63 (.37)

	Hare	62 (.36)	**106 (.63)**


For the purpose of evaluating the generalizability of our binary tree model, we determined its accuracy when applied to a separate set of holdout test data. As a first step, all data from the cross-validation training set was fitted into the final binary tree model with optimal hyperparameters, and then we predicted whether or not test set participants belonged to the unicorn group. Participants predicted as non-unicorns were assigned to the second binary model. Using the second binary model, we were able to predict whether those non-unicorns belonged to the tortoise or hare group. Based on the results presented in Table 2, Model 1 was able to detect 5 out of 8 participants in the unicorn group (model accuracy = .74 over chance level .50). Model 2 correctly identified 57% of the non-unicorn group as belonging to the tortoise or hare groups (model accuracy = .59 over chance level .50). Overall, in the test set, after applying the final binary tree model, about 51% participants’ learning patterns were correctly predicted over chance level (33%).

**Table 2:** Confusion Matrix of Binary Tree Model Performance on Test Set.


	MODEL1 (N = 95)	PREDICTED CLASS	

		UNICORN	NON-UNICORN

True class	Unicorn	**5 (.63)**	3 (.37)

	Non-Unicorn	14 (.16)	**73 (.84)**

	**MODEL 2 (N = 76)**	**TORTOISE**	**HARE**

True class	Tortoise	**21 (.57)**	16 (.43)

	Hare	14 (.39)	**22 (.61)**

	Unicorn	2 (.67)	1 (.33)


### Predictor importance

**Figure 3:**
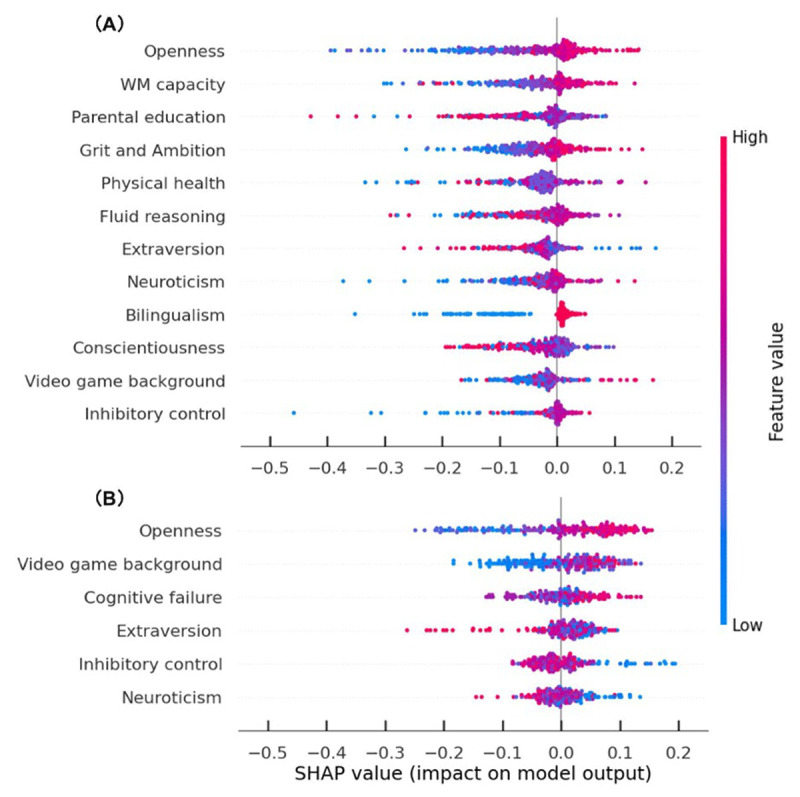
Predictor Importance by Calculating the SHAP Value. *Note*: The x-axis represents the SHAP value; The y-axis represents the predictors. Each dot represents each participant who is correctly predicted by our model (including both hitting a particular class and correctly rejecting the class), the color of the dot represents a certain predictor’s value (red: higher value, blue: lower value). **A.** Each predictor’s SHAP value in predicting the unicorn group (model 1) ranking by predictor importance. **B.** Each predictor’s SHAP value in predicting the tortoise group (model 2) ranking by predictor importance.

To interpret our final binary models, we calculated the mean SHAP value for each predictor to represent its feature impact (See Figure 3). Considering that the cross-validation set contains more data points than the test set, we examined the SHAP values of the cross-validation set. In addition, to focus on how the model successfully made predictions, the following visualizations only included those instances in the cross-validation set that were correctly predicted by our final models. A positive value indicates a positive contribution, that is, an increase of the probability of this person belonging to a particular class, while negative values indicate a decrease of the probability of belonging to that class.

**Figure 4:**
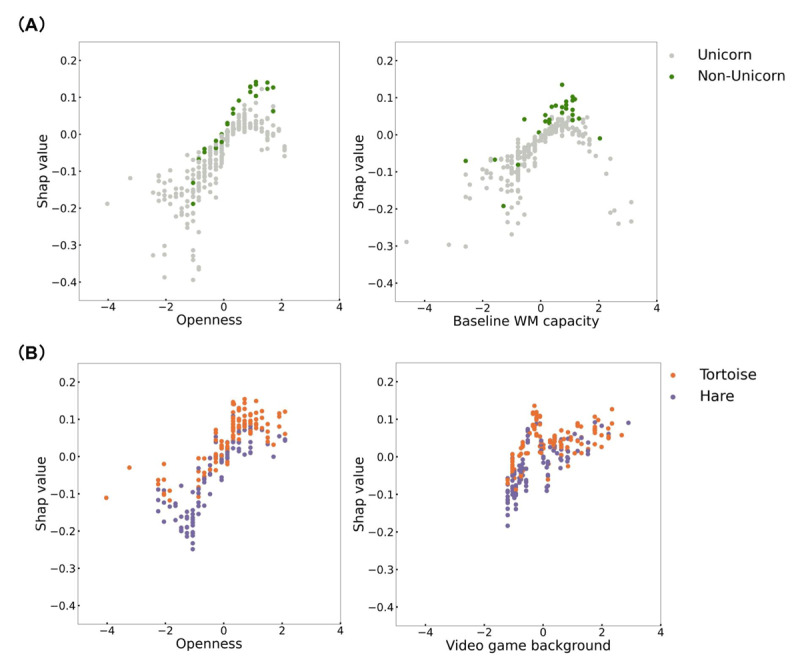
Scatter Plots of Predictor Values (x axis) and SHAP Values (y axis). *Note*: All predictor values were transformed to z scores. The top two important predictors in predicting unicorns (green) versus non-unicorns (gray) (Figure 4A) and tortoises (orange) versus hares (purple) (Figure 4B) are shown.

Figure 3A indicates the absolute mean of the SHAP values in predicting the unicorn group. Overall, openness and baseline WM capacity were the two most important factors contributing to the prediction. Specifically, participants who score higher in openness and with better baseline WM capacity were more likely to be classified as the unicorn group. Interestingly, openness and video game background emerged as the top two factors when predicting tortoise or hare groups (Figure 3B). Participants with a higher value of openness and more experience with video games were predicted to belong to the tortoise group. Figure 4 provides the scatter plots illustrating the relationship between the most important features values and their SHAP values.

## Discussion

Our study used machine learning models to examine whether and how a combination of individual difference variables could predict inter-individual variations in learning trajectories in the context of WM training. We used a clustering approach to identify three different participant groups (“Unicorns”, “Tortoises”, and “Hares”) relying on their early learning slopes and turning points during WM skill learning. The unicorns demonstrated high initial performance and quick learning, while the tortoises and hares displayed lower performance initially, with gradual improvements over time. Tortoises and hares differed in that tortoises continued to improve over longer periods, ending up with a relatively higher performance, whereas hares’ performance leveled off earlier. We then developed a binary tree model to predict which learning patterns participants belonged to. Among our 18 individual difference predictors, openness, as well as baseline WM capacity were the highest contributing factors in the first binary model (RBF support vector classifier) when predicting whether or not participants were unicorns. When we generalized the first model to new samples, our predictive accuracy was .74. For the Non-Unicorn group, we applied a second binary model (random forest classifier) to predict whether they were hares or tortoises. In this second model, we found that openness and video game background were the most important factors, and the predictive power for the new samples was .59.

The three different learning trajectories that emerged in our analyses broadly support Ackerman’s theory (2007) in demonstrating that differences between individuals become more pronounced with training. Additionally, the observed differences among the three patterns strongly support the magnification account (especially when comparing the unicorn group and non-unicorn group), with individuals initially demonstrating higher performance showing the most pronounced training gains, while those with lower initial performance exhibiting markedly less improvement (Lövdén et al., 2012; Rebok et al., 2007). This finding is consistent with a recent study by Ørskov et al. (2021) that used a similar intervention (dual-n-back training) in an adolescent population. Several studies, including Ørskov et al., argue that strategy use might explain this magnification effect (Fellman et al., 2020; Könen & Karbach, 2015). According to those authors, high performers might be better at coming up with effective strategies for specific tasks, which leads to faster learning and better overall performance. An alternative account suggests that higher initial cognitive abilities may also determine the later stages of skill learning (Taatgen, 2001). That is, high performers are better at learning in general, which translates to steeper training slopes, which we observe in our data. Laine et al. (2018) argued that those who are more successful at WM training are likely to use training task-related strategies such as updating and grouping, thus, participants need to heavily rely on their task-related WM resources, especially at higher N levels. As such, if the magnification effect was driven by strategy use, we should have found that participants’ baseline WM updating as measured by untrained n-back tasks to be among the highest contributing factors in predicting whether participants were high performers. Instead, our results showed that more general WM capacity (measured by several WM tasks) carried the most weight in the prediction, indicating that more general WM abilities seem to contribute more to learning than task specific performance and/or strategies, which is more in line with Ackerman’s theory (Ackerman, 1992). In addition to testing the prediction power of specific task performance, we also tested the prediction power of training-related processes based only on the initial performance within the training tasks themselves. However, it turned out that initial training performance did not have a good predictive performance (see Table S8), indicating that the more general factors we collected before training were more powerful predictors than the task-specific performance at the beginning of training, again, supporting the notion that any learnt strategies extend beyond the trained task, which is important because this might ultimately explain transfer (e.g. Pahor et al., 2022).

Consistent with our hypothesis, the training trajectories of participants were found to be best predicted by a combination of individual difference variables, including personality characteristics. Specifically, we observed a significant positive correlation between feature value and SHAP value, indicating that individuals with higher levels of openness were more likely to be predicted as unicorns. Openness is characterized by traits such as open-mindedness, creativity, and insightfulness. Individuals high in openness tend to embrace variety and diversity, as well as to be curious about their surroundings, and adopt creative approaches when faced with changing circumstances (McCrae & Sutin, 2009; Sutin, 2017). Such adaptive traits have been found to be positively correlated with deep learning instead of surface learning (Chamorro-Premuzic & Furnham, 2009). Consistent with this perspective, a recent study of a one-year long cognitive intervention in older adults demonstrates a greater improvement in learning among individuals with more adaptive personality traits (Kekäläinen et al., 2023). Interestingly, openness predicts the tortoise in a positive linear manner as well. It may indicate that open-mindedness and creativity are crucial elements for completing a relatively extensive training program with adaptive learning challenges. This might manifest in trying out different strategies to figure out the optimal one, and/or being more open to the idea that cognitive training might be beneficial, which might lead to more persistence. In addition, we noticed a trend indicating that participants with more video game experience were classified as «tortoises» who persisted longer throughout the intervention as compared to “hares” (Figure 4B). This aligns with previous research highlighting the cognitive benefits of video gaming (Steyvers & Schafer, 2020; Waris et al., 2019; Zhang et al., 2021), in particular, the ‘learning to learn’ framework (Bavelier et al., 2012). The repetitive nature of video games may also cultivate patience for cognitive tasks, enabling individuals to maintain their performance over time.

When using SHAP values to explain how the different factors predict learning patterns, we found that the impact of factors on learning trajectories is non-linear in most cases. As shown in Figure 4A, there is an inverted «U» shaped relationship between baseline WM capacity and its contribution to predicting unicorns. Importantly, this pattern confirms that individual differences can have non-linear effects on training performance, and machine learning is an effective tool to uncover these nonlinear relationships. Most participants in the unicorn group demonstrated higher baseline WM capacity compared to the average. However, it should be noted that individuals with the highest WM capacity were not necessarily classified as part of the unicorn group. This further demonstrates that a participant’s training performance is determined by a combination of variables. Although we provide a ranking of predictor importance in Figure 3, this does not necessarily imply that the most significant factors explain the full variance. It is important to note though that even in our relatively large sample for psychology field, the unicorn group was the smallest in terms of sample size (n = 45), and thus, typical intervention studies that have fewer participants might not include enough of such super learners or they might just see one single participant like this and might exclude them as an outlier. The use of machine learning enables us to detect and predict subgroups, which is a significant advantage over traditional regression analysis. At the same time, our findings highlight the importance of looking at subgroups when determining their contributing factors to learning outcomes, which requires large-enough sample sizes.

Overall, our study took a novel approach to exploring individual differences in WM training by using machine learning, which allowed us to go beyond explaining the linear relationship between one or few separate individual-difference variables and learning outcomes. At the same time, we acknowledge several limitations. First, despite the fact that our sample size was larger than that of most cognitive training studies (Bogg, & Lasecki, 2015), it was still small as compared to other studies that implement machine learning models (Youyou et al., 2015). In order to minimize the risk of overfitting, we used oversampling, feature selection strategies, and most importantly, a separate test set. Despite these strategies, our results might not generalize to the entire population. Since machine learning models using psychological experimental data are still rare, this study served first and foremost as a preliminary exploration. Second, our study used indicators from piecewise linear regression to describe training trajectories (Ørskov et al., 2021). Learning patterns that rely on other indicators might result in a different prediction model. Indeed, completely data-driven approaches are highly dependent on the type of data provided, and choosing the most appropriate learning pattern can be challenging. Third, due to time constraints, certain predictors, such as physical health, were measured using one item. This limited assessment may not fully capture the comprehensive representation of the predictor. Lastly, our combination of individual difference variables did not have a high level of predictive power. There are likely other factors that were not captured by our assessments, or there is unmodelable uncertainty that creates variation in the data.

Despite these limitations, our observation that baseline cognitive abilities are effective predictors of learning patterns is consistent with the existing literature and theory (e.g. Ackerman, 1992; Karbach et al., 2017). Furthermore, our study illustrates that different combinations of individual difference variables can be used to distinguish between different types of learning patterns. This implies that the relationship between individual differences and WM training gains is complex, and that the most important factors may vary across different types of individuals. This may also explain why the results of previous studies have been inconsistent and further highlights the need to take individual differences into account, which requires large sample sizes. By identifying these predictors, we can potentially identify sub-populations that are more amenable to targeted interventions. Notably, we also observed a few participants who did not show any performance changes during training, which has often been overlooked in previous studies. Although the sample size was limited, we found that those participants’ fluid reasoning performance was either extremely high or low (see Figure S4). Participants with extremely low reasoning performance may be less well suited for this type of cognitive training, while those with extremely high reasoning performance may find the training boring, resulting in a lack of engagement. However, it is worth noting that any task design (and gamification) will be inherently be more welcoming to some participants and less welcoming to others and so future research is needed to better address aspects of task design may be needed to reach different populations rather than concluding that some populations are less well suited for particular tasks. On the basis of this study, future research can develop more advanced models for predicting individual training trajectories or transfer effects over large datasets, which can ultimately inform the design of more effective and productive training programs. Such training programs could be then used to identify at-risk students according to individual differences and in turn, provide refined and personalized interventions, which is the purpose of precision education (Luan & Tsai, 2021).

## Data Accessibility Statements

The data that support the findings of this study are openly available in OSF at https://osf.io/yuza3/.

## Additional File

The additional file for this article can be found as follows:

10.5334/joc.319.s1 Supplementary Materials. Supplementary Figures s1 to s4 and Tables S1 to s9.

## Footnotes

Only 3 participants were over the age of 40. We decided to include them because excluding/leaving them in the sample did not change the results.

We also collected participants’ cognitive assessments after training, however, they are not included for the purpose of the present paper. These data are reported elsewhere (e.g. Pahor et al., 2021; 2022).
