# Supplementary material for: Unicorn, Hare, or Tortoise? Using Machine Learning to Predict Working Memory Training Performance
Source: J Cogn. 2023 Sep 4;6(1):53. doi: 10.5334/joc.319 (PMC10487130; doi:10.5334/joc.319)
Supplement: Supplementary Materials. — Supplementary Figures s1 to s4 and Tables S1 to s9. [file joc-6-1-319-s1.pdf]

Supplementary Materials for

Unicorn, Hare, or Tortoise? Using Machine Learning To Predict Working Memory Training Performance

Yi Feng[1], Anja Pahor[2,4,5], Aaron R. Seitz[2,4], Dennis L. Barbour[3] & Susanne M. Jaeggi[1,4]

[1]University of California, Irvine, School of Education, School of Social Sciences (Department of Cognitive Sciences), Irvine, California, USA

[2]University of California, Riverside, Department of Psychology, Riverside, California, USA

[3]Washington University in St. Louis, Department of Biomedical Engineering, St. Louis, Missouri, USA

[4]Northeastern University, Department of Psychology, Boston, Massachusetts, USA

[5]University of Maribor, Department of Psychology, Maribor, Slovenia

Corresponding author: Yi Feng, yif12@uci.edu

Training Task

Figure S1

Non-gamified and Gamified Variants of the N-back Training Tasks

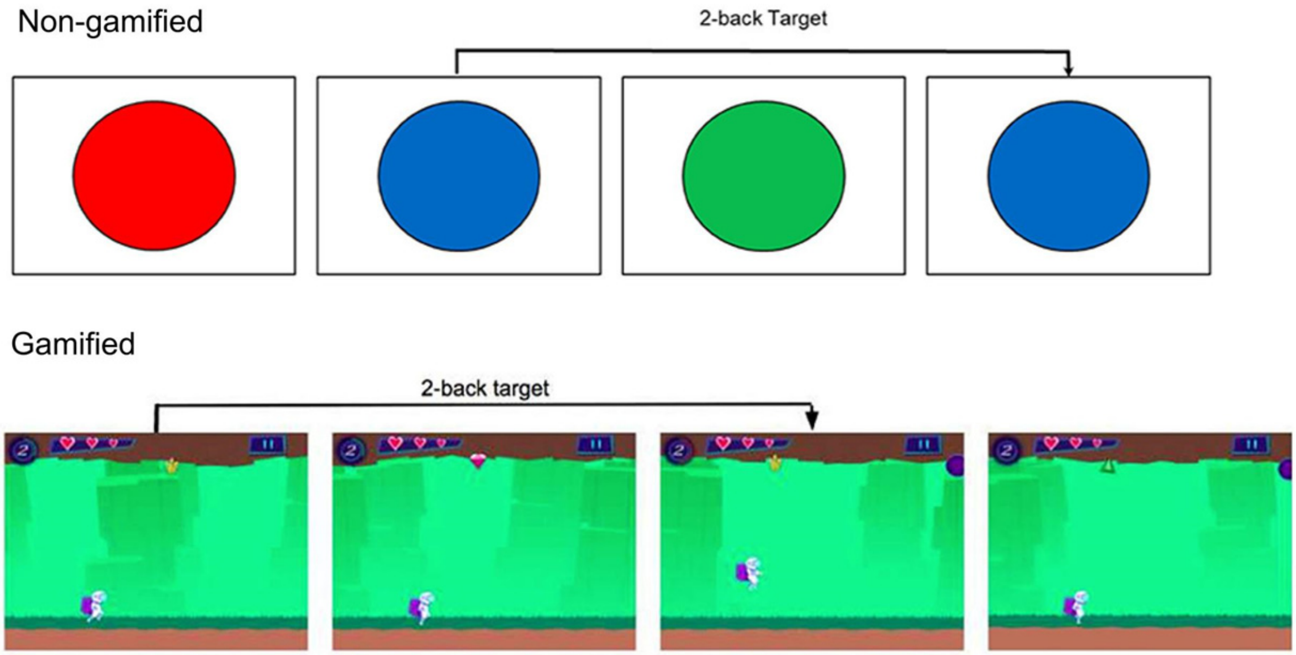

Note. The training paradigm was similar as described in Sandeep et al., 2020.

Predictors descriptive information

Table S1

Descriptive Information for All Predictors, Along with Correlation Coefficients

Predictor variables	N	IR	Mean	SD	Min	Max	Kurt.	Skew.	1	2	3	4	5	6	7	8	9	10	11	12	13	14	15	16	17
**Cognitive assessments**																									
1. WM Updating	565	0.2	-0.04	0.79	-2.23	2.17	-0.27	-0.09																	
2. WM capacity	566	0.2	-0.04	0.75	-3.62	2.37	1.96	-0.21	.21**																
3. Inhibitory control	567	0	0.00	0.92	-6.98	2.87	15.36	-2.35	.02	.044															
4. Fluid reasoning	567	0.2	.61	.28	0	1	-0.25	-0.54	.28**	.22**	.02														
**Person-Related factors- Self-report measures**																									
5. Growth mindset	220	/	20.02	4.54	6	30	-0.02	-0.38	.08	.08	.06	-.03													
6. Cognitive failure	527	0	24.19	5.42	8	40	-0.02	0.12	.10*	.04	-.01	.10*	-.01												
7. Grit and Ambition	528	0	3.60	0.57	1.91	5	-0.29	-0.21	-.05	.00	-.13*	-.18**	.24**	-.35**											
8. Neuroticism	527	0	3.37	0.70	1.5	4.88	-0.57	-0.08	.00	.09*	.03	-.08	.12	-.31**	.29**										
9. Extraversion	527	0	3.10	0.77	1.38	5	-0.60	-0.10	-.04	.05	-.01	-.09*	.11	-.15**	.38**	.10*									
10. Openness	527	0	3.69	0.62	1.13	5	0.28	-0.37	.07	-.01	-.01	-.05	.09	.03	.33**	-.02	.35**								
11. Agreeable.	527	0	4.05	0.59	1.63	5	0.54	-0.76	.06	.00	.04	-.03	.28**	-.16**	.33**	.28**	.29**	.29**							
12. Conscious.	527	0	3.72	0.65	1.75	5	-0.13	-0.46	.00	-.02	-.04	-.10*	.16*	-.34**	.56**	.22**	.20**	.24**	.31**						
**Environmental Factors and Experience**																									
13. Parents' education	508	5.6	4.75	1.31	1	7	-0.29	-0.29	.06	-.01	-.04	-.07	.02	-.21**	.19**	.22**	.13*	.07	.12*	.18**					
14. Self-reported SES	529	0.2	11.19	2.87	4	20	0.17	0.04	-.10*	-.09*	.06	-.02	-.06	.13*	-.24**	-.15**	-.24**	-.19**	-.20**	-.16**	-.20**				
15. Video game.	526	0.2	0.01	0.99	-1.16	4.00	0.06	0.87	.11*	.10*	.00	.19**	-.01	.06	-.13*	-.00	-.06	.00	-.10	-.16**	.00	-.06			
16. Physical health	490	9.2	3.25	0.74	1	5	0.54	0.37	.07	-.01	-.10	-.09*	.05	-.08	.21**	.07	.21**	.16**	.08	.11*	.19**	-.28**	-.09*		
17. Psycho. health	490	9.2	3.46	0.89	1	5	-0.43	-0.11	.04	.05	-.01	-.10*	.02	-.21**	.23**	.39**	.30**	.04	.17	.13*	.31**	-.26**	.02	.26**	
18. Physical fitness	490	9.2	3.06	0.91	1	5	-0.31	0.04	.06	.05	-.09	-.03	.10	-.13*	.33**	.13*	.30**	.19**	.15	.21**	.22**	-.29**	-.11*	.65**	.28**

Note. IR = Imputation Rate (%); Kurt. = Kurtosis; Skew. = Skewness
Agreeable = Agreeableness; Conscious. = Consciousness; Video game. = Video game background; Psycho. = Psychological
* = p < .05, ** = p < .01

Three predictors utilized a composite score derived from different measures. The correlation between these measures and their respective composite scores is reported as follows: 1) WM Updating: The accuracy of the 2-back task showed a significant correlation with the accuracy of the 3-back task (*r* = .314**). The composite z-score exhibited a strong correlation with 2-back accuracy (*r* = .826**) and 3-back accuracy (*r* = .795**). 2) WM Capacity: WM capacity was measured using the Letter-Number task and the Corsi Block Forward task, which exhibited a correlation of .131**. The composite z-score demonstrated a high correlation with Letter-Number span (*r* = .745**) and Corsi Block Forward span (*r* = .759**). 3) Grit and Ambition: Ambition was commonly associated with grit in our dataset, displaying a correlation of .518. The composite score of grit and ambition showed a strong correlation with ambition (*r* = .897**) and grit (*r* = .843**).

Training performance

Figure S2

Clustering Results with Different K Numbers

(A)

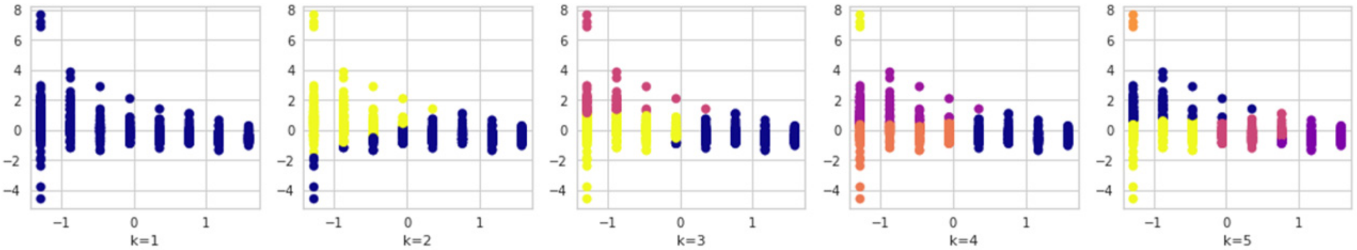

(B)

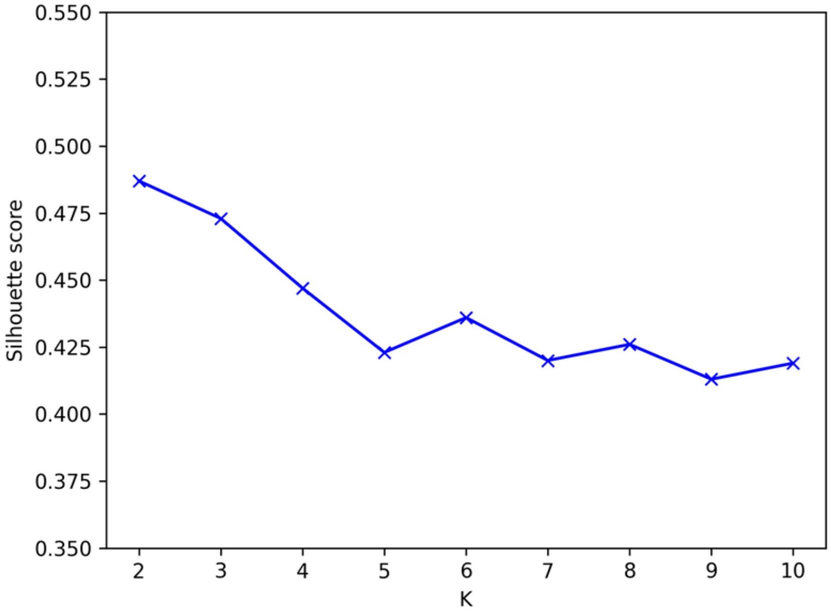

Figure S3

Training Trajectories by Cluster

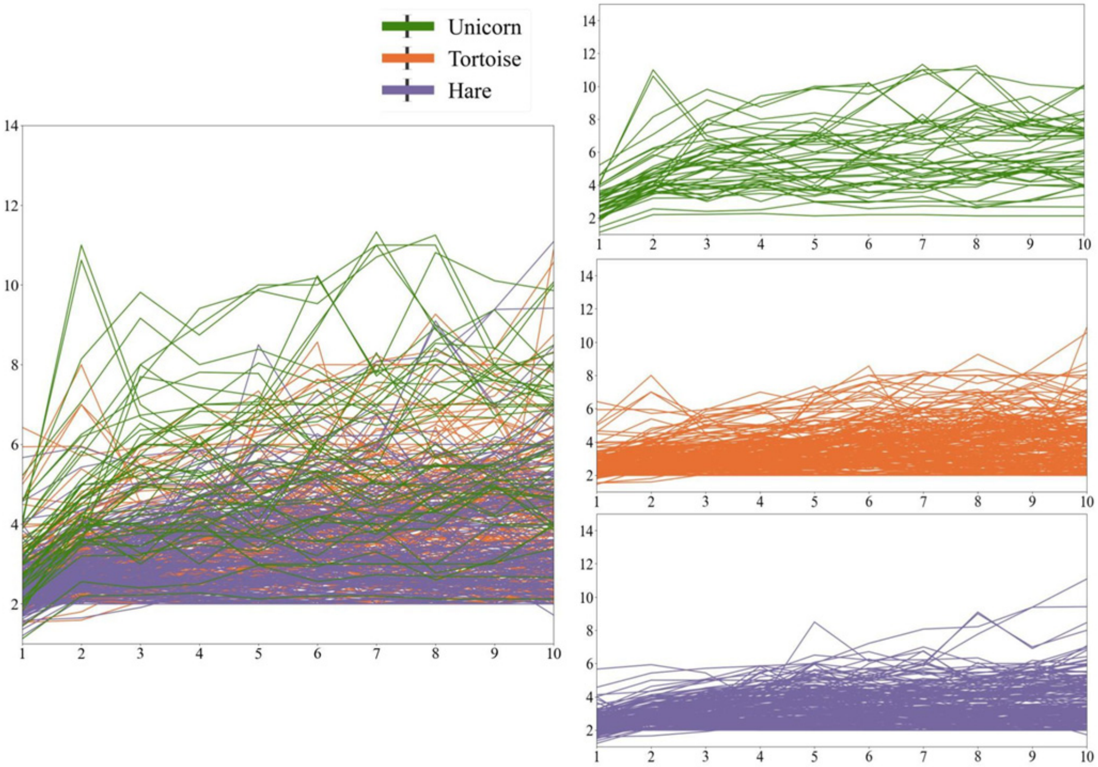

Participants that showed no change during training

In our sample, there were 31 participants who did not show any performance change over the course of the training. We referred to this group as the "starfish group" given their lack of movement. Among them, 27 participants consistently performed at the 2-back level in each training session, while the remaining 4 participants consistently performed at the 3-back level. In order to investigate whether there are predictors that can distinguish between participants in the starfish group and those in the non-starfish group, we trained a binary model following the same model training steps as described in the main text. The results revealed that the RBF Support Vector Model performed the best on the cross-validation set (Table S2), with an average accuracy of .94 (*SD* = .02). However, the test set showed poor accuracy (.17) since there were only 6 starfish participants in the test set. Still, we calculated the SHAP values in the cross-validation set to get some insight of which predictors might be able to distinguish between the starfish and non-starfish group. The results showed that fluid reasoning was the dominant predictor in the model (mean |SHAP value| = .21), followed by video game background as the second important predictor (mean |SHAP value| = .05). Interestingly, most of the participants who showed no performance change during training showed either extremely high or low performance on the fluid reasoning task (cf. Figure S4, and Table S3), suggesting that the reason for the lack of learning might be either related to motivation (e.g., those with high scores in fluid intelligence) or related to ability and/or motivation (those with low scores in fluid intelligence).

Table S2

Confusion Matrix: Model Performance in Detecting Participants with No Change during Training (Cross-Validation Set)

		Predicted class	
	Model (N=401)	Starfish	Non-Starfish
True class	Starfish	**24 (1.00)**	0 (.00)
	Non-Starfish	31 (.08)	**346 (.92)**

Figure S4

Scatter Plot of Fluid Reasoning and its SHAP Values

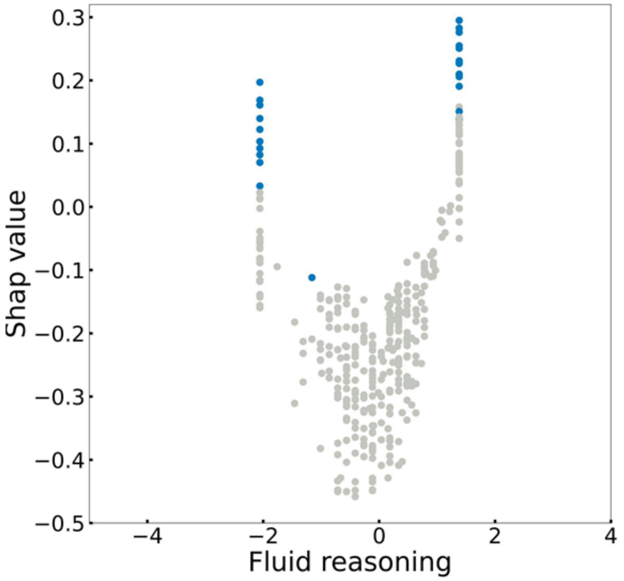

Note. x-axis: Fluid reasoning standardized score; y-axis: SHAP values. Blue dots: Starfish group who did not show any performance change during training; Gray dots: non-starfish group.

Table S3

Frequency Table of Starfish Group's Fluid Reasoning

	N	Fluid Reasoning (Accuracy)
Consistent at 2-back	12	0
	1	.217
	2	.261
	1	.619
	11	1
Consistent at 3-back	1	0
	3	1

Exploration of regression models

Since we had a continuous distribution of participants' learning slope rather than distinct clusters, we also trained regression models to directly predict their learning slope. The results of different models showed similar performance, see Table S4. The results demonstrated that these regression models exhibited a limited predictive power.

Table S4

Regression Model Performance (test set)

Model	MAE	MSE	RMSE
Linear Regression	.292	.133	.365
RBF Support Vector Model	.272	.128	.358
Decision Tree	.285	.128	.359
Random Forest	.291	.130	.360
KNeighbors Regressor	.348	.202	.450
Multilayer Perceptron Regressor	.322	.182	.426

Note. MAE: Mean Absolute Error; MSE: Mean Squared Error; RMSE: Root Mean Squared Error.

Model performance with all features

To assess the necessity of using feature selection, we trained a binary tree model using all predictors, excluding the training program information. The results are shown in Table S5. The performance of Model 1 was slightly better than the performance with feature selection, while Model 2 showed a slightly lower performance, indicating that the additional features did not improve the model's predictive power.

Table S5

Confusion matrix of binary tree model with all features (cross-validation set)

		Predicted class	
	Model1 (N=377)	Unicorn	Non-Unicorn
True class	Unicorn	**28 (.88)**	4 (.39)
	Non-Unicorn	43 (.31)	**302 (.88)**
	Model2 (N=368)	Tortoise	Hare
True class	Tortoise	**109 (.62)**	68 (.38)
	Hare	64 (.38)	**104 (.62)**

Classification model selection

We evaluated multiple classification models during the model selection phase, utilizing both linear and non-linear algorithms. The performance of these models on the cross-validation set is presented in Table S6. For the first model, our objective was to detect unicorns, as they exhibited excellent learning performance. In this case, the repercussions of missing positive instances were considered to be more significant than the consequences of false positives. As a result, the selection of model 1 was based on both recall (to maximize the detection of unicorns) and accuracy. On the other hand, model 2 was developed to differentiate between hares and tortoises. In this scenario, our primary focus was on accuracy, aiming to correctly classify as many instances as possible. Therefore, the selection of model 2 was primarily based on accuracy. By considering the goals and characteristics of each model, we chose the RBF Support Vector Model for model 1 and the Random Forest model for model 2.

Table S6

Model Performance of Different Classification Algorithms (cross-validation set)

Model 1	Precision	Recall	F1 score	Mean Accuracy (SD)
Logistic Regression	.129	.562	.209	.681 (.050)
Decision Tree	.264	.594	.365	.869 (.044)
Random Forest	.568	.656	.609	.954 (.018)
Linear Support Vector Model	.129	.594	.212	.690 (.065)
RBF Support Vector Model	.311	.875	.459	.902 (.029)
Multiple Layer Perceptron	.286	.688	.404	.898 (.031)
Model 2				
Logistic Regression	.581	.525	.552	.556 (.041)
Decision Tree	.586	.576	.581	.580 (.066)
Random Forest	.648	.644	.646	.643 (.052)
Linear Support Vector Model	.604	.542	.571	.575 (.056)
RBF Support Vector Model	.594	.571	.582	.581 (.053)
Multiple Layer Perceptron	.588	.548	.567	.571 (.054)

$$\text{Precision} = \frac{\text{[illegible]}}{\text{[illegible]} + \text{[illegible]}}$$

$$\text{Recall} = \frac{\text{[illegible]}}{\text{[illegible]} + \text{[illegible]}}$$

$$\text{F1 score} = \frac{\text{[illegible]}}{\text{[illegible]} + \frac{1}{2}(\text{[illegible]} + \text{[illegible]})}$$

Training conditions as predictors

Table S7

Model Performance After Adding Training Program Features (cross-validation set)

Model	Precision	Recall	F1 score	Mean Accuracy
Original model 1	.311	.875	.459	.902
model 1 + Training conditions	.358	.750	.485	.813
model 1 + Training conditions + Gamified/non-Gamified	.359	.719	.479	.800
Original model 2	.648	.644	.646	.643
model 2 + Training conditions	.664	.616	.639	.645
model 2 + Training conditions + Gamified/non-Gamified	.663	.644	.653	.649

Note. Training conditions represent 10 different algorithms.

Another approach to predict learning patterns

Even though our updating task that we used as a baseline measure was an untrained variant of the N-back task used during training, it might not capture the same baseline ability as the training task itself. Therefore, we considered using the initial performance on the training task itself to predict learning patterns. Given that this was only one predictor, we applied a simple logistic regression model with exactly the same dataset. The confusion matrix is shown in Table S8. In the first model, the F1 score is .256, and in the second model, it is .565.

Table S8

Confusion matrix of the logistic model with one predictor (cross-validation set)

		Predicted class	
	Model1 (N=404)	Unicorn	Non-Unicorn
True class	Unicorn	**22 (.61)**	14 (.39)
	Non-Unicorn	114 (.31)	**254 (.69)**
	Model2 (N=368)	Tortoise	Hare
True class	Tortoise	**98 (.52)**	90 (.48)
	Hare	61 (.34)	**119 (.66)**

Cohort-based descriptions of learning pattern groups

Table S9 displays the frequencies of each learning pattern group by cohorts. The results indicate that there was no learning pattern specific to any particular cohort.

Table S9

Distribution of Learning Pattern Groups Across Cohorts

Cohort	Total N	Unicorn	Tortoise	Hare	Starfish	2-back algorithm	Outliers
1	266	25	103	114	1	11	12
2	138	7	57	43	27	/	4
3	164	13	76	68	3	/	4

Note. The "2-back algorithm" category represents participants from a specific algorithm that solely implemented the 2-back task. Therefore, this category was excluded from our analysis.
